# Glutathione *S*-conjugates as prodrugs to target drug-resistant tumors

**DOI:** 10.3389/fphar.2014.00181

**Published:** 2014-08-11

**Authors:** Emma E. Ramsay, Pierre J. Dilda

**Affiliations:** Tumour Metabolism Group, Adult Cancer Program, Lowy Cancer Research Centre and Prince of Wales Clinical School, Faculty of Medicine, University of New South WalesSydney, NSW, Australia

**Keywords:** glutathione *S*-conjugate, prodrug, cancer, drug resistance, glutathione transferase, gamma-glutamyl transferase

## Abstract

Living organisms are continuously exposed to xenobiotics. The major phase of enzymatic detoxification in many species is the conjugation of activated xenobiotics to reduced glutathione (GSH) catalyzed by the glutathione-*S*-transferase (GST). It has been reported that some compounds, once transformed into glutathione *S*-conjugates, enter the mercapturic acid pathway whose end products are highly reactive and toxic for the cell responsible for their production. The cytotoxicity of these GSH conjugates depends essentially on GST and gamma-glutamyl transferases (γGT), the enzymes which initiate the mercapturic acid synthesis pathway. Numerous studies support the view that the expression of GST and γGT in cancer cells represents an important factor in the appearance of a more aggressive and resistant phenotype. High levels of tumor GST and γGT expression were employed to selectively target tumor with GST- or γGT-activated drugs. This strategy, explored over the last two decades, has recently been successful using GST-activated nitrogen mustard (TLK286) and γGT-activated arsenic-based (GSAO and Darinaparsin) prodrugs confirming the potential of GSH-conjugates as anticancer drugs.

## INTRODUCTION

Glutathione (GSH) plays a myriad of roles in the body. It is a major cellular antioxidant, involved in defense against oxidative stress and redox signaling. GSH also modulates cell proliferation, apoptosis, immune function, and fibrogenesis (Lu, 2013). In cancer cells, GSH and enzymes of the mercapturic acid pathway play a role in resistance to many chemotherapeutic drugs. However, not all xenobiotics (including drugs) are conjugated/inactivated by this pathway (Commandeur et al., 1995); some are in fact activated into cytotoxic compounds. This review explores the importance of the mercapturic acid pathway and the potential of intra-tumor activation of old and recently discovered chemotherapeutics. The expression patterns of participating enzymes of the mercapturic acid pathway could theoretically be employed to drive the activation of such compounds within the tumor.

**FIGURE 1:**
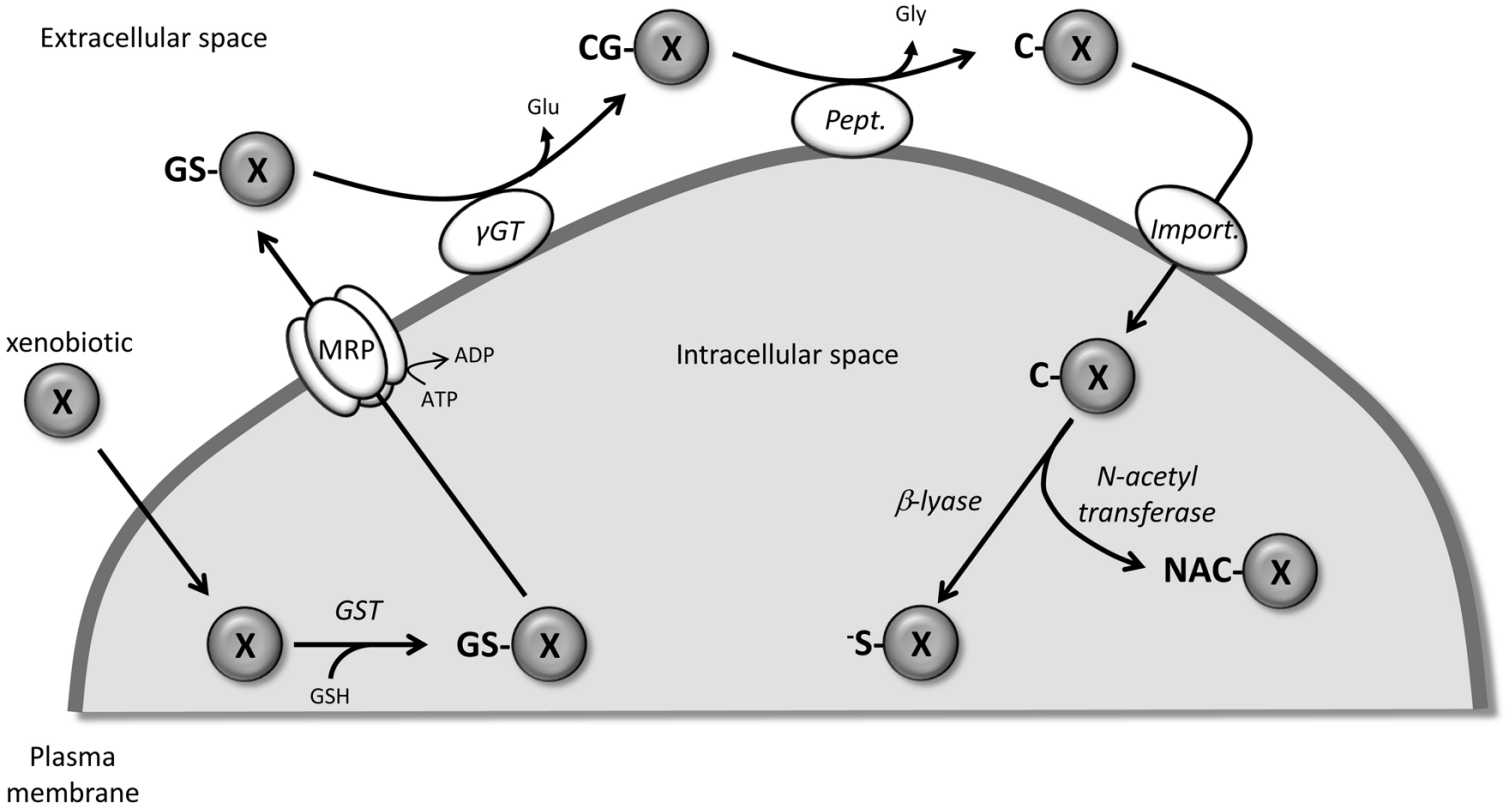
**Detoxification of xenobiotic through the mercapturic acid synthesis pathway.** X, xenobiotic; GS-X, GSH-conjugated version of X; CG-X, cysteinylglycyl-X; C-X, cysteinyl-X; NAC-X, mercapturic acid version of X; ^-^S-X, highly reactive thiol version of X; GST, glutathione *S*-transferase, γGT, gamma-glutamyl transferase; Pept., peptidase; Import., peptide transporter or cystine/cysteine importer.

The mercapturic acid pathway (**Figure 1**) involves the conjugation of the tripeptide, GSH, to xenobiotics (including drugs) to render them more hydrophilic and facilitate their elimination. Although some spontaneous reactions could occur, the cytosolic glutathione transferases (GST) catalyze the nucleophilic conjugation of GSH with a wide spectrum of electrophiles (Armstrong, 1997). Being the first step in the metabolism and eventual removal of the drug from the body, GST is associated with chemotherapeutic resistance since exposure to these drugs is often associated with induction of GST, especially GST P1-1 (Dahllof et al., 1987; Hamada et al., 1994; Hao et al., 1994). The GSH-conjugated compounds generated are then actively pumped out of the cell by numerous members of the MRP/ABCC family, which appear to have broad and partially overlapping substrate specificity (Ballatori et al., 2009). At the cell surface, the first step in the catabolism of GSH-conjugates is catalyzed by the membrane-bound gamma-glutamyl transferase (γGT) which removes the GSH γ-glutamyl group and transfers it to appropriate acceptors. Similar to what has been described for GST, γGT is also considered to be part of a resistance phenotype. The main reason for this is the role that γGT plays in maintaining appropriate GSH levels in the cells for xenobiotic detoxification by conjugation (Pompella et al., 2006). The newly formed cysteinylglycine *S*-conjugates are further processed by dipeptidases/aminopeptidases to remove the glycyl group and produce cysteine *S*-conjugates. These compounds then re-enter the cell via various transporters including organic anion transport polypeptides and cystine/cysteine importers (Hinchman et al., 1998; Budy et al., 2006; Dilda et al., 2008, 2009; Garnier et al., 2014). In the cytosol, *N*-acetyl transferases create mercapturic acid versions of the xenobiotics which are generally more polar and more water soluble than the parental compound. At this point, the compounds are generally non-toxic and excreted from the body through bile or urine. Alternatively, instead of acetylation, some compounds can be converted by cysteine *S*-conjugate β-lyase to produce an unstable and highly reactive thiol.

There are several examples of drugs becoming toxic/bioactive following their conjugation with GSH. Trichloroethylene is metabolized to form [*S*-(1,2-dichlorovinyl)GSH], which is the first step in the GSH metabolism pathway. *In vitro* (Lash et al., 1986; Chen et al., 1990; Cummings and Lash, 2000) and *in vivo* (Terracini and Parker, 1965; Elfarra et al., 1986), the downstream metabolites of GSH *S*-dichlorovinyl, such as *N*-acetyl-*S*-(1,2-dichlorvinyl)-L-cysteine and *S*-(1,2-dichlorovinyl)-L-cysteine, induce toxicity towards the cells responsible for their production (Spencer and Schaumburg, 1985; Patel et al., 1993). Consistent with what was observed with trichloroethylene, the GSH-*S*-conjugate of 4-hydroxynonenal (HNE) causes a loss in cell viability in γGT-expressing cells (Enoiu et al., 2002). This effect was attributed to the cysteinylglycine *S*-conjugate of HNE (Calonghi et al., 2002; Cerbone et al., 2007; Pettazzoni et al., 2011).

The expression of several enzymes involved in the mercapturic acid pathway (notably GST and γGT) is induced in cancer cells and particularly drug resistant cancer cells (**Table 1**). The specific expression of these enzymes involved in the local production of cytotoxic/bioactive drugs has been exploited to design and develop various types of anticancer prodrugs. This review describes the enzymes involved in drug activation in the order they participate along the mercapturic acid pathway, GST, γGT, and β-lyase. The potential to exploit the induced expression of these enzymes as predictive markers and the opportunity this presents for drug design is discussed.

**Table 1:** Activity and expression levels of glutathione-*S*-transferase and gamma-glutamyl transferase in tumor tissues.

Tumor	Glutathione *S*-transferase		Gamma-glutamyl transferase	
	Activity or expression vs. normal tissue	Reference	Expression in tumor tissue	Reference
Liver	↓ 0.50	Howie et al. (1990)	++	Tsutsumi et al. (1996), Hanigan et al. (1999a)


	↓ Pol. M1, T1	Song et al. (2012)		

Kidney	↓ 0.27–0.44	Di Ilio et al. (1987), Wang et al. (1989)	++	Hanigan et al. (1999a)
	↓ Pol. P1, M1, T1	Ahmad et al. (2012)	↑ vs. normal tissue (activity)	Arai et al. (1995)

Esophagus	↑ 5.75	Tsuchida et al. (1989)	+	Hanigan et al. (1999a)
	↓ Expression P1	Wang et al. (2010)	↑ vs. normal tissue (expression and activity)	Fiala et al. (1980)

Stomach	↑ 1.36–1.66	Howie et al. (1990), Peters et al. (1990)	±	Hanigan et al. (1999a)

Colon	↑ 1.37–4.3	Tsuchida et al. (1989), Moorghen et al. (1991)	+	Hanigan et al. (1999a)

			↑ vs. normal tissue (expression and activity)	Fiala et al. (1980), Murata et al. (1997)

Pancreas	↓ Pol. M1, T1	Fan et al. (2013)	+	Hanigan et al. (1999a), Ramsay et al. (2014)

Lung	↑ 1.83–2.63	Di Ilio et al. (1987), Howie et al. (1990)	±	Hanigan et al. (1999a)

			↑ vs. normal tissue (expression)	Tateishi et al. (1976), Dempo et al. (1981), Blair et al. (1997)

Thyroid	↓ Pol. P1, M1, T1	Gaspar et al. (2004), Li et al. (2012)	+	Hanigan et al. (1999a)

Breast	→ 0.85–1.33	Forrester et al. (1990),Howie et al. (1990)	++	Durham et al. (1997), Hanigan et al. (1999a),
	↓ Pol., methyl. P1	Saxena et al. (2012)	↑ vs. normal tissue (expression and activity)	Fiala et al. (1980), Bard et al. (1986)

Ovary	↑ Expression	Hetland et al. (2012)	+	Paolicchi et al. (1996), Hanigan et al. (1999a, 1994), Grimm et al. (2013)

Prostate	↓ Pol. P1, M1, T1	Moskaluk et al. (1997), Rebbeck et al. (1999)	++	Hanigan et al. (1999a)
	↓ Methyl. P1	Esteller et al. (1998)	↑ vs. normal tissue (expression and activity)	Fiala et al. (1980)

Urinary bladder	↑ 5.17	Lafuente et al. (1990)	↑ vs normal tissue (expression and activity)	Fiala et al. (1980)

	→ Expression	Oğuztüzün et al. (2011)		

*For GST, arrows indicate if the measured activity or expression is increased (↑), decreased (↓) or stable (→) in comparison with normal tissue. Values are the ratio of GST activity in tumor tissue with GST activity in normal tissue. Pol. refers to polymorphism of GST genes responsible for the loss of activity of GST P1 (P1), GST M1 (M1), or GST T1 (T1) isoenzymes. Methyl. refers to DNA methylation in the promoter region of GSTP1 gene responsible for epigenetic silencing of this isoenzyme. For gamma-glutamyl transferase, arrows indicate that the measured activity or expression is increased (↑) in comparison with normal tissue. The signs + and ± indicate the level of γGT expression in tumor tissues measured by immunohistochemistry.*

## BIOACTIVATION OF CANCER COMPOUNDS BY GST

Glutathione transferases (EC 2.5.1.18) are a superfamily of dimeric detoxification enzymes which contribute to the cellular biotransformation of electrophilic compounds (Mannervik et al., 2005). They provide protection against genotoxic and carcinogenic effects of numerous substances of both xenobiotic and endogenous origins. The essential role of GST is to catalyze the conjugation of GSH with a wide variety of compounds, including drugs, resulting in the formation of the corresponding GSH-conjugates and subsequently facilitate their clearance from the body. In humans, various isoforms of GST are present in virtually all tissues with the liver exhibiting the highest cytosolic GST activity level followed by kidney, lung, and intestine (Pacifici et al., 1988).

### GST AND CANCER

Although GST detoxifying activity protects cells from endogenous toxic products, it also blunts the effectiveness of certain anticancer drugs (O’Brien and Tew, 1996). GST and GSH are frequently elevated in many tumors relative to surrounding healthy tissue (Howie et al., 1990). Isoenzyme GST P1-1 is notably induced in lung, colon, and stomach cancers and was found to be implicated in cellular resistance to chemotherapeutic agents (Dahllof et al., 1987; Hamada et al., 1994; Hao et al., 1994).

The polymorphism in GSTM1 and GSTT1 genes, leads to complete lack of activity of their corresponding enzymes and is responsible for poor elimination of carcinogenic substances which are potential sources of reactive oxygen species (Rebbeck, 1997; Kumar et al., 2011). This polymorphism has been associated with increased risk of benign prostatic hyperplasia and prostate cancer (Rebbeck et al., 1999) and can be a useful biomarker to identify patients at higher risk for fatal prostate cancer (Agalliu et al., 2006; Liu et al., 2013). In prostate, but also breast, endometrial and hepatocellular carcinomas, another early tumor marker is GSTP1 promoter methylation which is detected at various percentages of clinical samples (Esteller et al., 1998; Zhong et al., 2002; Chan et al., 2005; Lee, 2007; Yoon et al., 2012). The epigenetic silencing of GSTP1 provides a mechanism of resistance which makes tumors with GSTP1 promoter methylation bad candidates for the GST-activated anti-cancer prodrugs presented in this review. However, recent studies demonstrated that it is possible to re-sensitize tumors using a combination of molecules specifically reverting the aberrant DNA methylation in cancer cells (through drugs inhibiting DNA methyltransferase activity) and cytotoxic drugs (Plumb et al., 2000; Cheng et al., 2003; Sabatino et al., 2013).

### GST ANTICANCER PRODRUGS

An approach to solving drug resistance due to over expression of GST is to design specific GST inhibitors (Ruzza et al., 2009). Alternatively, GST overexpression offers the opportunity to target resistant tumors with GST-activated prodrugs. This type of compound undergoes GST-catalyzed breakdown to release locally cytotoxic metabolites and thereby attenuate off target adverse side effects. Amongst these prodrugs, two categories can be distinguished.

**FIGURE 2:**
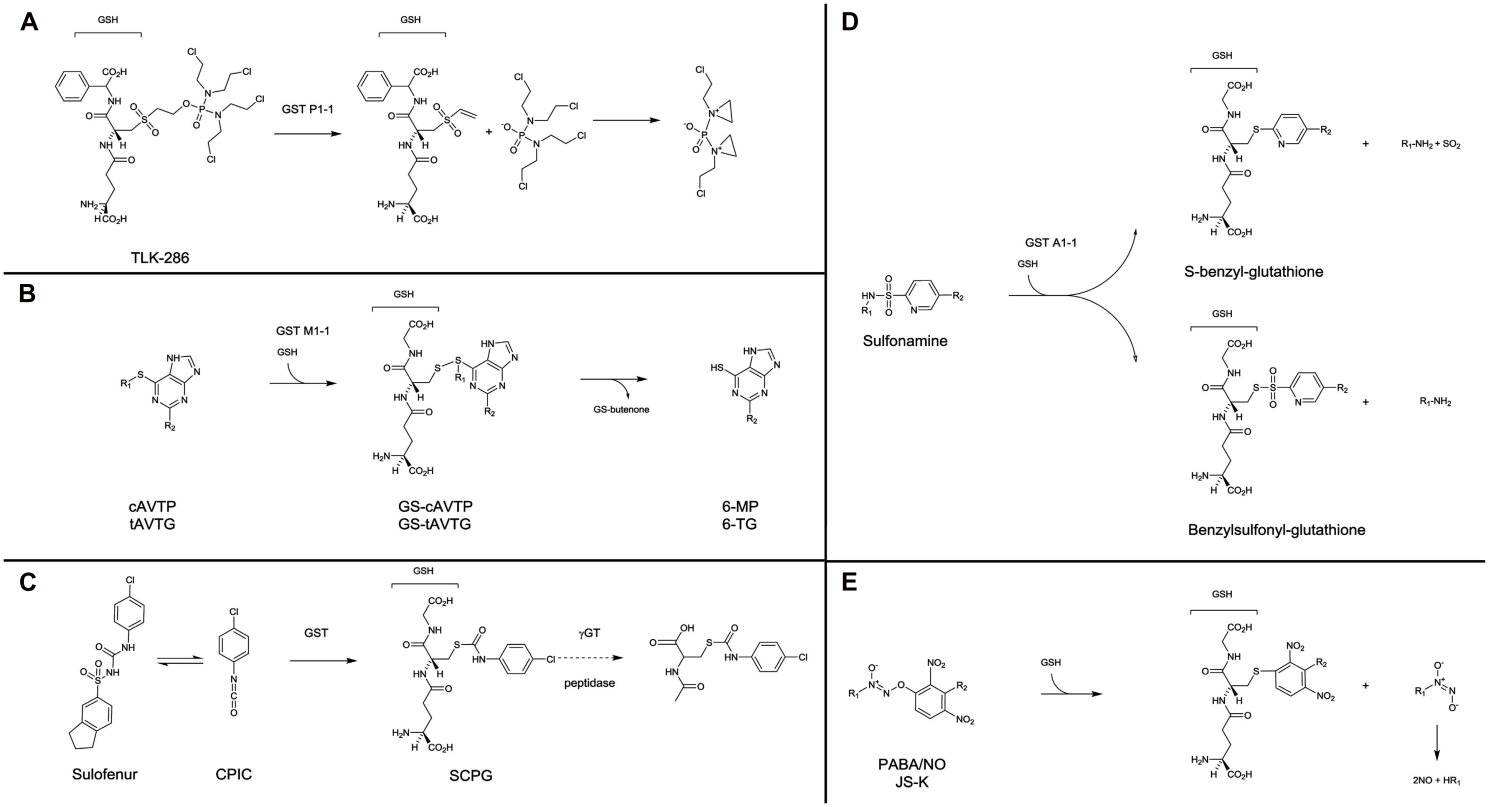
**Mechanisms of activation of GST prodrugs.** TLK286 **(A)** has a GSH-like moiety in its structure, in contrast, all other presented drugs are conjugated with GSH during their process of activation by GST. **(B)** for cAVTP, GS-cAVTP, and 6-MP, R_1_: *cis*-butenone; R_2_: H. For tAVTG, GS-tAVTG and 6-TG, R_1_: *trans*-butenone; R_2_: NH_2_. **(D)** for sulfonamine compounds, R_1_: bombesin or aryl derivatives; R_2_: CN or CF_3_. **(E)** for PABA/NO, R_1_: NMe2; R_2_: 4-carboxy-*N*-methylanilinyl. For JS-K, R_1_: 1-carboxyethyl-4 piperazinyl, R_2_: H.

The first category consists of prodrugs containing a GSH or GSH-like structure for which a β-elimination reaction, catalyzed by GST, releases a cytotoxic compound. This includes compounds such as nitrogen mustards, which have the property to alkylate DNA after further transformation: TLK286 (canfosfamide, TELCYTA^TM^) and analogs (Lyttle et al., 1994; Satyam et al., 1996; Dourado et al., 2013; **Figure 2A**). TLK286 showed interesting *in vitro* and *in vivo* antiproliferative activity on cells with high GST P1-1 expression as a result of selection for doxorubicin, cyclophosphamide, and platinum resistance (Morgan et al., 1998; Townsend et al., 2002). In murine xenografts, tumor growth inhibition, or regression in response to TLK286 was positively correlated with the level of GST P1-1 expression (Morgan et al., 1998). Several clinical trials demonstrated that TLK286 was active and safe to use in combination treatment regimens with standard chemotherapeutic agents, including platinums, taxanes, and anthracyclines. Noteworthy, clinical efficacy was observed with both relapsed patients with ovarian and non-small cell lung cancers, and in the first-line treatment setting in non-small cell lung cancer patients (Sequist et al., 2009; Vergote et al., 2010).

The second category regroups prodrugs non-structurally related to GSH. However, their activation by GST involves the production of an intermediate GSH-conjugate.

– In this category, *cis*-6-(2-acetylvinylthio)-purine (cAVTP) and *trans*-6-(2-acetylvinylthio)-guanine (tAVTG) contain active thiopurines (6-mercaptopurine and 6-thioguanine, respectively) liberated intracellularly after GSH-conjugation catalyzed by GST M1-1, M2-2, and A4-4, but not GST P1-1 (Gunnarsdottir et al., 2002; Eklund et al., 2007; **Figure 2B**). Both compounds exhibited a remarkable growth-inhibitory activity towards leukemic and melanoma cells (Gunnarsdottir and Elfarra, 2004). *In vivo*, on the contrary to what was observed following the administration of bioactive end products (6-mercaptopurine and 6-thioguanine), no reduction of circulating white blood cells was observed upon administration of cAVTP and tAVTG (Gunnarsdottir et al., 2002).
– Using a similar strategy, exocyclic enones displaying antitumor activity were produced by a GSH-dependent reaction catalyzed by GST (Hamilton et al., 2002, 2003). COMC-6 (2-crotonyloxymethyl-2-cyclohexenone) is a potent antitumor agent against both murine and human tumors in culture and in tumor-bearing mice (Aghil et al., 1992).
– Diarylsulfonylureas are a class of antitumor agent with interesting therapeutic efficacy against a wide range of cancers (Howbert et al., 1990; Mohamadi et al., 1992). Sulofenur [(*N*-(5-indanesulfonyl)-*N*′-(4-chlorophenyl)urea, LY18664], the prototypic member of this class (**Figure 2C**), was evaluated in clinical trials on a variety of patients with kidney (Mahjoubi et al., 1993), ovary (O’Brien et al., 1992), breast (Talbot et al., 1993), lung (Munshi et al., 1993), and stomach (Kamthan et al., 1992) cancers. It appeared that sulofenur can undergo metabolic biotransformation to yield the GSH-conjugate of *p*-chlorophenyl isocyanate [*S*-(*N*-*p*-chlorophenylcarbamoyl) GSH, SCPG; Jochheim et al., 2002]. Whether a direct involvement of GST is necessary or not in the production of this GSH-conjugate remains unclear. However, it was clearly demonstrated that SCPG is further processed through the mercapturic acid pathway to the corresponding N-acetylcysteine conjugate [*N*-acetyl-*S*-(*p*-chlorophenylcarbamoyl)cysteine, NACC], which possesses comparable anticancer activity to the parent compound. The GSH- and cysteine-conjugates produced are susceptible to thiol exchange reactions and may act as carbamoylating agents towards biomacromolecules (Day et al., 1996; Guan et al., 2002). NACC, whose mechanism of action remains unclear, demonstrated selective anticancer activity, and low toxicity, which make NACC and its analogs promising anticancer agents (Chen et al., 2011).
– Another approach was to use the sulphonamidase activity of GST. GST catalyzes the GSH-mediated hydrolysis of sulfonamide bonds, releasing GST competitive inhibitors such as benzylsulfonyl-GSH or *S*-benzyl-GSH (Koeplinger et al., 1999; Zhao et al., 1999; Axarli et al., 2009; **Figure 2D**).
– Finally, electrophilic diazenium diolates were designed as prodrugs for spontaneous nitric oxide (NO) release at physiological pH, after a reaction with GSH catalyzed by GST. Essentially two compounds were studied. PABA/NO [(*O*^2^-(2,4-dinitro-5-(*N*-methyl-*N*-4 carboxyphenylamino)phenyl) 1-*N*,*N*-dimethylamino)diazen-1-ium-1,2-diolate; Findlay et al., 2004] and JS-K [*O*^2^-(2,4-dinitrophenyl) 1-[(4-ethoxycarbonyl) piperazin-1-yl]diazen-1-ium-1,2-diolate; Shami et al., 2003] were selectively activated by GST M1 and GST A1, respectively, and produced abundant NO in the tumor and contributed to chemotherapy by GSH consumption, DNA synthesis inhibition and inhibition of enzymes responsible for cellular damage repair (**Figure 2E**). PABA/NO and JS-K demonstrated antitumor activity in mice bearing human ovarian carcinoma (Findlay et al., 2004) and prostate carcinoma (Laschak et al., 2012) or multiple myeloma (Kiziltepe et al., 2007), respectively. Recently, new generations of more stable and potent GST-activated NO prodrugs have been developed (Liu et al., 2012; Fu et al., 2013).

## GLUTATHIONE *S*-DERIVATIVES ACTIVATED BY γGT AND PEPTIDASES

Gamma-glutamyl transferase (EC2.3.2.2) catalyzes the transpeptidation and hydrolysis of the γ-glutamyl group of GSH and related compounds to an acceptor molecule, including water, amino acids, and peptides. It is found on the plasma membrane, facing extracellularly, playing an essential role in the maintenance of intracellular cysteine (Moriarty-Craige and Jones, 2004). Considering GSH’s important role as a cellular antioxidant, γGT has traditionally been considered a component of the cell’s oxidative stress defenses. However, the range of γGT substrates has expanded considerably, now including a range of GSH conjugates, leukotriene C4 (LTC4), *S*-nitroso-GSH and GSH adducts of xenobiotics, suggesting diverse roles for γGT.

High expression of γGT is commonly found on cells involved in transport, on the luminal surface of secretory and absorptive cells. Its highest expression is on the luminal surface of the proximal tubules in the kidney, whilst the bile ducts, bile canaliculi, and endothelial cells of the nervous system capillaries also have high expression (Shiozawa et al., 1989; Hanigan and Frierson, 1996).

Oxidative stress has been shown to induce γGT expression (Kugelman et al., 1994; Knickelbein et al., 1996; Liu et al., 1998; Borud et al., 2000; Roomi et al., 2006). High γGT expression protects melanoma cells from hydrogen peroxide or ascorbic acid induced oxidative stress (Giommarelli et al., 2008). It also allows the cells to maintain their intracellular GSH levels and subsequently respond to oxidative stress. Conversely, γGT has been demonstrated to have pro-oxidant effects. Combined with metal ions (iron or copper), γGT can induce lipid peroxidation (, ; Stark and Glass, 1997). γGT has been linked to reactive oxygen species generation in cells (Drozdz et al., 1998; Del Bello et al., 1999; Paolicchi et al., 2002).

### γGT AND CANCER

Gamma-glutamyl transferases expression has been shown to be increased in numerous cancers. Increased levels have been observed in cancer of the ovary, liver, lung, and breast, and in melanoma and leukemia (Fujisawa et al., 1976; Gerber and Thung, 1980; Dempo et al., 1981; Bard et al., 1986; Corti et al., 2010; Mareš et al., 2012). In many cases the γGT levels are higher in the corresponding primary tumor (Maellaro et al., 2000). An extensive study by Hanigan et al. (1999a) scored the expression of γGT in a variety of tumors. Carcinomas in particular express γGT, with carcinomas of the kidney, liver, and prostate showing strong expression (Hanigan et al., 1999a). Furthermore, some carcinomas of the breast, ovary, uterus, and pancreas were shown to express γGT. On the contrary, non-epithelial malignancies and sarcomas rarely expressed γGT.

Gamma-glutamyl transferases has been considered an early marker of neoplastic transformation. Many early studies have demonstrated in *in vivo* models the appearance of γGT expression in areas previously negative following exposure to carcinogens (Pompella et al., 2006). The underlying mechanism of this phenomenon remains unclear. A genome-wide analysis of pancreatic cancer implicated GGT1 as playing a role in carcinogenesis (Diergaarde et al., 2010). The proto-oncogene KRAS was shown to be involved in the upregulation of γGT expression. Recently, Moon et al. (2012) have demonstrated that KRAS transformed prostate epithelial cells are more resistant to hydrogen peroxide induced free-radicals than non-transformed cells. They observed an upregulation of GGT2 in the KRAS transfected cells and confirmed its role in resistance to hydrogen peroxide treatment. This has also been observed in colon carcinoma cells where radiation induced γGT activity was mediated through Ras pathway (Pankiv et al., 2006).

The early appearance of γGT in neoplasms suggests the potential for γGT to play a role in tumor progression. γGT has been shown to give cells a growth advantage *in vitro* and *in vivo*. High expression of γGT provides cells with greater quantities of cysteine through the breakdown of extracellular GSH (Gerber and Thung, 1980; Hanigan, 1995; Hochwald et al., 1996). This explains the difference in growth rates of cells when moved from the *in vitro* setting to mice, where extracellular GSH and cysteine is limited *in vivo*. In clones of melanoma cells, the extent of γGT expression was shown to be proportional to the invasive ability of the clone (Supino et al., 1992).

### γGT AND DRUG RESISTANCE

Beyond its differential expression in cancer, γGT is considered to be part of a resistance phenotype (Pompella et al., 2006). The main reason for this is the role that γGT plays in maintaining GSH levels within the cell. GSH plays an important part in the detoxification of xenobiotics by binding to a range of agents and this allows for GSH mediated expulsion of these compounds from the cell.

The relationship between γGT and chemotherapy resistance is demonstrated by a number of experiments showing that transfection with γGT both *in vitro* and *in vivo* leads to resistance to members of the platinum drug family, and in particular to *cisplatin* (Hanigan et al., 1999b; Daubeuf et al., 2002; Franzini et al., 2006). Further evidence of the relationship comes from biopsies from ovarian adenocarcinoma patients before and after treatment (cisplatin, chlorambucil, and 5FU). Cells grown from biopsies taken before and after treatment, showed a 6.5-fold increase in γGT activity following treatment (Lewis et al., 1988). However, Schäfer et al. (2001) found no direct link between γGT and resistance, despite an evident growth advantage for γGT overexpressing cells.

Another proposed mechanism for platinum drug resistance is the formation of adducts between the platinum drug and the cysteinyl-glycine product of γGT. These complexes have poor ability to cross the cell membrane; as a result the platinum drug rarely reaches its target (DNA). These adducts have been described in the extracellular media of γGT overexpressing cells and the plasma of patients treated with oxaliplatin (Daubeuf et al., 2002, 2003; Paolicchi et al., 2003; Jerremalm et al., 2006;Corti et al., 2010).

Gamma-glutamyl transferases has also been implicated in resistance to radiation therapy. In lymphoid cells, γGT plays a role in maintaining the intracellular GSH levels that are essential for protection against radiation (Jensen and Meister, 1983). Inhibition of γGT in melanoma cells significantly increased the radiosensitivity of a high γGT variant (Prezioso et al., 1994b). In CC531, a colon cancer cell line, γGT was upregulated in a time and dose-dependent manner to irradiation. This increase in γGT activity was attributed to *de novo* synthesis of the mRNA. It was further demonstrated that signaling through the Ras pathway was responsible for this increase (Pankiv et al., 2006).

### HIGH γGT EXPRESSION AND INTRATUMOR DRUG ACTIVATION

The high expression of γGT within aggressive and drug-resistant tumors implies that GSH-conjugated prodrugs, activated at the cell surface by γGT, should be particularly effective against γGT-positive tumors. The metabolism of these compounds by γGT positive tumor cells should produce high local concentrations of bioactive and membrane permeable metabolites that would then block tumor cell proliferation and eventually tumor growth. Increasing the concentration of active metabolites at the tumor site, will subsequently limit the concentration of active drug at other sites, reducing side effects.

#### Arsenic-based GSH-conjugates

GSAO [4-(*N*-(*S*-glutathionylacetyl)amino)phenylarsonous acid] and Darinaparsin (ZIO-101; *S*-dimethylarsino-GSH) are arsenic-based GSH-conjugates with demonstrated antitumor activity evaluated in clinical trials (Don et al., 2003; Dilda et al., 2005b; Diaz et al., 2008; Tsimberidou et al., 2009; Hosein et al., 2012; Horsley et al., 2013). For both drugs, extracellular γGT activity is an essential and limiting step in their activation into membrane permeable compounds (Dilda et al., 2008; Garnier et al., 2014). Indeed, it has recently been demonstrated that tumor γGT could be used for therapeutic delivery (Ramsay et al., 2014).

***GSAO.*** 4-(*N*-(*S*-glutathionylacetyl)amino)phenylarsonous acid is a prospective cancer drug and has just completed a phase I dose escalation study in patients with solid tumors refractory to standard therapy (Horsley et al., 2013). Treatment was very well tolerated. Of 34 patients, 20 were evaluated for response (receiving two or more cycles of GSAO). Whilst no patient exhibited an objective response, eight had stable disease, with one patient having stable disease for 18 weeks.

**FIGURE 3:**
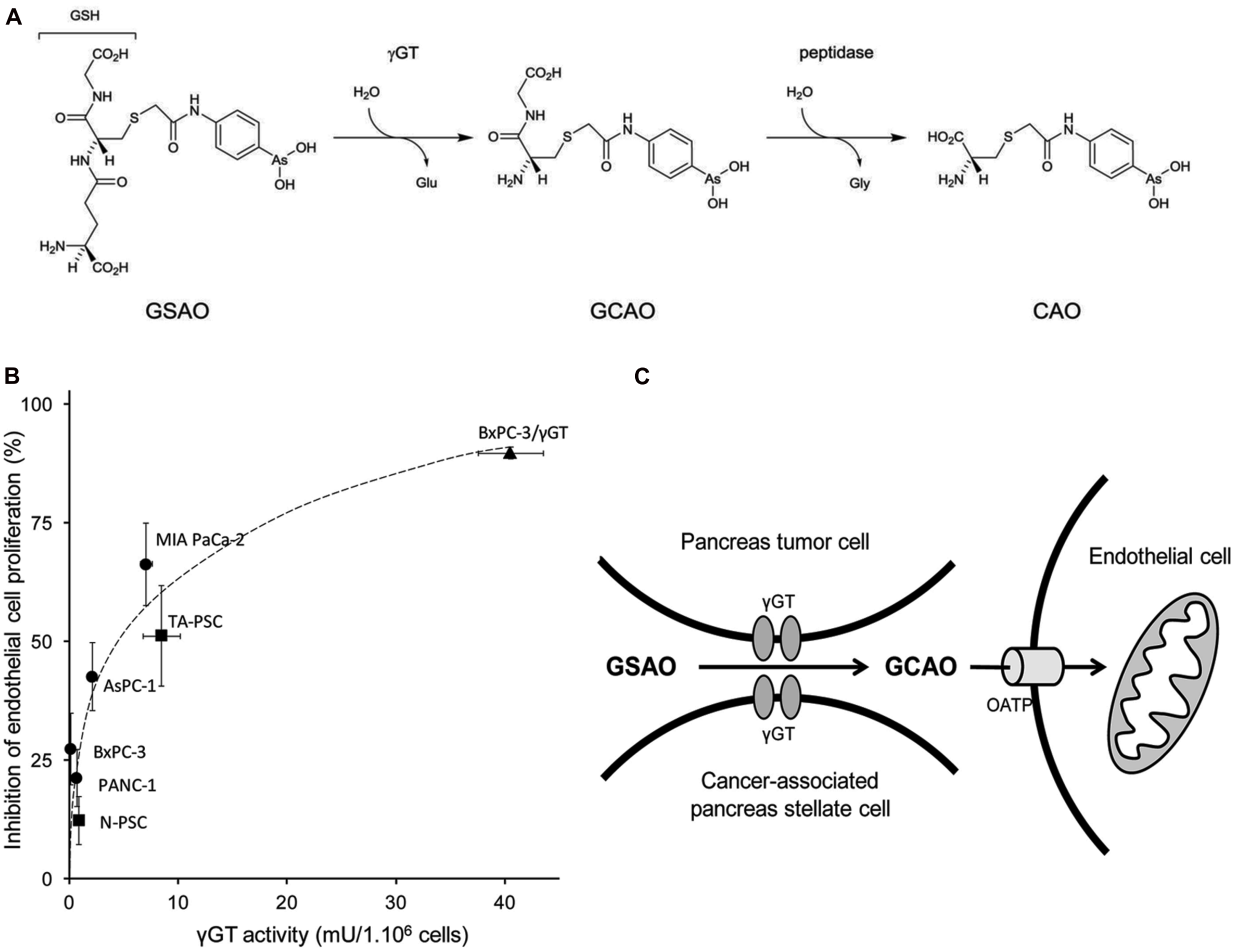
**Activation process of GSAO and subsequent activity on endothelial cell proliferation. (A)** GSAO activation by γGT and peptidase. **(B)** Pancreatic tumor cell γGT activity positively correlates with GSAO-mediated proliferation arrest of endothelial cells in a transwell model. Pancreatic adenocarcinoma cells are represented by circles and normal and tumor-activated pancreatic stellate cells by squares. BxPC-3 pancreatic tumor cells transfected with γGT are represented as triangles. **(C)** Model of GSAO activation by tumor γGT (from Ramsay et al., 2014).

4-(*N*-(*S*-glutathionylacetyl)amino)phenylarsonous acid consists of a phenylarsenoxide moiety attached by an *N*-acetyl linker to the cysteine thiol of reduced GSH (**Figure 3**). The phenylarsenoxide group is the active moiety, imparting to GSAO its activity by crosslinking closely spaced protein thiols and forming a high affinity ring structure between its arsenic and the thiols (Donoghue et al., 2000). GSAO specifically targets proliferative endothelial cells, which consequently starves the tumor of the nutrients required to support its expanding growth (Don et al., 2003; Dilda et al., 2005b). The GSH moiety contributes to the transport of GSAO in and out of the cell (Dilda et al., 2008, 2005b).

4-(*N*-(*S*-glutathionylacetyl)amino)phenylarsonous acid in its original form is essentially membrane impermeable. Upon reaching the cell surface the γ-glutamyl group is cleaved by γGT (Dilda et al., 2008), generating the dipeptide form, 4-(*N*-(*S*-cysteinylglycylacetyl) amino) phenylarsonous acid (GCAO). GCAO is then able to enter the cell through organic anion transporters (OATPs). Within the cell further processing by dipeptidases likely occurs, resulting in the formation of the single amino acid form of GSAO, 4-(*N*-(*S*-cysteinylacetyl)amino)phenylarsonous acid (CAO; Dilda et al., 2008; **Figure 3A**).

The phenylarsenoxide moiety of CAO has been confirmed to crosslink the cysteine residues 57 and 257 of the adenosine nucleotide translocator (ANT; Park et al., 2012). ANT is the most abundant protein found on the inner mitochondrial membrane and it is responsible for the exchange of matrix ATP for cytosolic ADP across the inner mitochondrial membrane. Disruption of its function has been shown to have major impacts on mitochondrial integrity and cell survival (Halestrap et al., 2002; McStay et al., 2002; Don et al., 2003). The calcium-dependent binding of CAO to ANT induces a conformational change (Halestrap et al., 2002) which results in the opening of the mitochondrial permeability transition pore (MPTP) and allows the equilibration of small solutes and the release of pro-apoptotic proteins from the intermembrane space. The equilibration that results leads to a collapse of the proton-motive force across the membrane and a colloid osmotic pressure that causes massive swelling of the mitochondria (McStay et al., 2002).

There are a number of factors that influence GSAO activity: the level of expression of the enzyme responsible for its activation (γGT); intracellular GSH levels; and the expression of the multi-drug resistance association proteins, ABCC1 (MRP1) and ABCC2 (MRP2). The combination of low intracellular GSH levels and low expression of ABCC1 and ABCC2 in proliferative endothelial cells accounts for GSAO selectivity and subsequently for GSAO anti-angiogenic properties (Dilda et al., 2005a,b; Park and Dilda, 2010).

Pancreatic tumors display the most prominent stromal/desmoplastic reaction of all epithelial tumors (Apte and Wilson, 2012). Knowing that pancreatic tumorigenesis has been associated with expression of γGT by both cancer and tumor-associated stellate cells (Ramsay et al., 2014), Ramsay et al. (2014) have explored the utility of this enzyme in delivering GSAO to pancreatic ductal adenocarcinoma. They demonstrated that human pancreatic tumor and stellate cells activate/process GSAO into its active metabolite and that γGT activity positively correlates with GSAO-mediated proliferation arrest of co-cultured endothelial cells (**Figure 3B**). Importantly, tumor γGT activity positively correlates with GSAO-mediated inhibition of pancreatic tumor angiogenesis and tumor growth in mice.

4-(*N*-(*S*-penicillaminylacetyl)-amino)phenylarsonous acid (PENAO), a cysteine mimetic analog of the cysteine-*S*-conjugate metabolite of GSAO has been investigated. In this compound, penicillamine replaces the cysteine moiety of CAO. PENAO was shown to have the same molecular target as GSAO (Dilda et al., 2009; Park et al., 2012). By bypassing the cell surface processing of GSAO, PENAO accumulated 85 times faster in cells (Dilda et al., 2009). This corresponds to an increase in anti-proliferative capacity on a variety of endothelial and cancer cell lines. Interestingly, PENAO has a strong anti-proliferative activity against glioblastoma cell lines (Chung et al., 2011) and primary isolates of diffuse intrinsic pontine glioma (Tsoli et al., 2013). *In vivo*, PENAO demonstrated preclinical activity without signs of toxicity in tumor models of glioblastoma (Chung et al., 2013) and pancreatic carcinoma (Dilda et al., 2009). In the later model, PENAO was approximately 20-fold more efficacious than GSAO (Dilda et al., 2009). Large scale animal toxicity studies demonstrated that PENAO was as well tolerated as GSAO, suggesting an interesting therapeutic window for this compound. PENAO is currently being tested in a clinical Phase I trial in patient with solid tumors refractory to standard chemotherapy. PENAO is another example of a mercapturic acid pathway metabolite with antitumor properties. However, the lack of processing by γGT removes the targeting advantage presented by GSAO.

***Darinaparsin.*** *S*-dimethylarsino-GSH (Darinaparsin; ZIO-101) is an organic arsenical compound currently in clinical development. It has been tested in Human with hematologic malignancies (Hosein et al., 2012; Nielsen et al., 2013) as well as solid tumors (Tsimberidou et al., 2009; Wu et al., 2010). Significantly more potent and better tolerated than arsenic trioxide, Darinaparsin showed encouraging responses in T-cell lymphoma (Hosein et al., 2012) and AML (Nielsen et al., 2013) patients. However, in a phase II evaluation of the compound in hepatocellular carcinoma, no objective response was shown (Wu et al., 2010).

**FIGURE 4:**
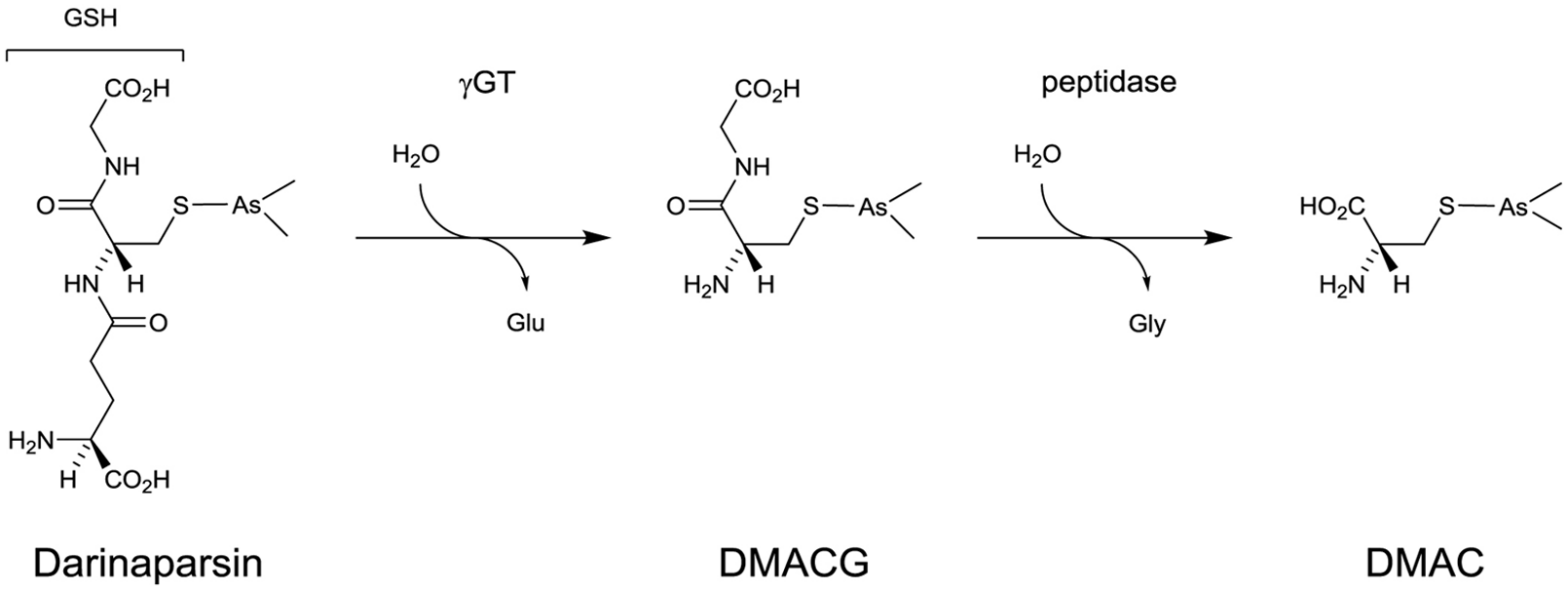
**Activation process of Darinaparsin**.

Darinaparsin belongs to the class of organic arsenical compounds that are generally considered less toxic than inorganic ones (Waxman and Anderson, 2001; Dilda and Hogg, 2007). It was synthesized by conjugating GSH to dimethyl arsenic (**Figure 4**). Darinaparsin shares some characteristics with other arsenicals but has also unique properties. As do other arsenicals, it induces G_2_/M cell cycle arrest and triggers apoptosis through disruption of mitochondrial functions and JNK activation and is responsible for reactive oxygen species production (Diaz et al., 2008). However, unlike arsenic trioxide, GSAO (Dilda et al., 2005b) or PENAO (Dilda et al., 2009), Darinaparsin activity is unaffected by the expression of ABCC1 (MRP-1), modulation of GSH levels (Diaz et al., 2008) and heme-oxygenase inhibition (Garnier et al., 2013).

Darinaparsin was characterized *in vitro* as a potent inducer of growth arrest and apoptosis in a range of hematologic malignancies such as acute promyelocytic leukemia (APL), acute lymphoblastic leukemia, B cell lymphoma, and multiple myeloma (Diaz et al., 2008; Matulis et al., 2009). In contrast to arsenic trioxide (Lallemand-Breitenbach et al., 2012) and like PENAO and melarsoprol (Chen et al., 2003), Darinaparsin, at concentrations that induce apoptosis, does not induce PML/RAR degradation in APL cells. In the context of solid cancer, Darinaparsin was found to have preferential cytotoxic and radiosensitizing effects as compared with normal cells both *in vitro* and in a clinically relevant model (Tian et al., 2012, 2013).

It was recently demonstrated that Darinaparsin, which is essentially a GSH *S*-conjugate of dimethylarsenic, needs to be processed at the cell surface before exerting its activity on various cancer cells. The GSH moiety of the drug has to be processed by γGT before further processing by dipeptidases to generate dimethylarsino-cysteine (DMAC), a cysteine *S*-conjugate which is imported via cystine transporters (Garnier et al., 2014; **Figure 4**). Whether DMAC could be subsequently transformed intracellularly into (i) a highly reactive and cytotoxic thiol through β-lyase activity or (ii) dimethylarsenite/dimethylarsenate is not known. Similarly to what was observed in the case of GSAO and CAO (Dilda et al., 2008), Garnier et al. (2014) demonstrated that DMAC recapitulates the effects of Darinaparsin and that γGT is the first and limiting step in the activation of the drug. They also linked Darinaparsin efficacy to the level of expression of cystine/cysteine importing systems in cancer cells.

#### S-nitrosoglutathione

*S*-nitrosoglutathione (GSNO) is the *S*-nitrosated derivative of GSH and is thought to be a critical mediator of the downstream signaling effects of NO (Broniowska et al., 2013). GSNO plays roles as a carrier for NO and in protein *S*-nitrosation or *S*-glutathiolation (Broniowska et al., 2013). GSNO can be cleaved by γGT (Hogg et al., 1997; Angeli et al., 2009; Bramanti et al., 2009), generating *S*-nitrosocysteinylglycine and releasing NO *in vitro*. GSNO has been tested in a number of clinical trials, the majority focusing on the cardiovascular effects of GSNO as a NO donor (Broniowska et al., 2013). As do other NO-donating compounds, GSNO induces oxidative stress and initiates apoptosis (Turchi, 2006). GSNO promotes NO-induced apoptosis in colon carcinoma cell lines through depletion of intracellular GSH and the release of NO, (Ho et al., 1999; Liu et al., 2003). In a variety of cell lines, GSNO treatment combined with a MEK inhibitor was shown to inhibit the proliferation and invasive phenotype of the cells (Furuhashi et al., 2012). In T and B lymphocytes the anti-proliferative effect of GSNO was dependent on γGT activity (Henson et al., 1999). The anti-proliferative activity of GSNO, combined with the overexpression of γGT in tumor cells, suggests the possibility of its utility in cancer therapy. This requires further understanding of the roles that NO plays in cancer.

In the light of the promising results obtained, notably with the arsenical-based GSH-conjugates, it appears that tumor γGT could potentially be employed in drug targeting and delivery. In addition to cleavage of the γ-glutamyl from a GSH moiety, γGT can also catalyze the cleavage of γ-glutamyl moieties from a variety of compounds. This property along with tissue/tumor expression of γGT was used to develop γ-glutamyl prodrugs.

#### γ-glutamyl conjugates and *N* acetyl γ-glutamyl conjugates

Due to the tissue distribution of γGT, γ-glutamyl prodrugs have been mostly developed for kidney applications. An anti-nociceptive prodrug, γ-glutamyl-dermorphin, was explored by comparing the pain threshold of mice (Misicka et al., 1996). More extensively investigated was the γ-glutamyl conjugate of L-DOPA (dihydroxyphenylalanine). This prodrug provides the precursor of dopamine, DOPA, upon activation and was explored as a potential renal vasodilator (Wilk et al., 1978; Worth et al., 1985; Sadiq et al., 2000). Clinical testing of the prodrug determined that despite reasonable kidney specificity it had low bioavailability (Lee, 1990). A number of other compounds were conjugated to a glutamyl group and tested; however none were approved for use (Huttunen and Rautio, 2011).

**FIGURE 5:**
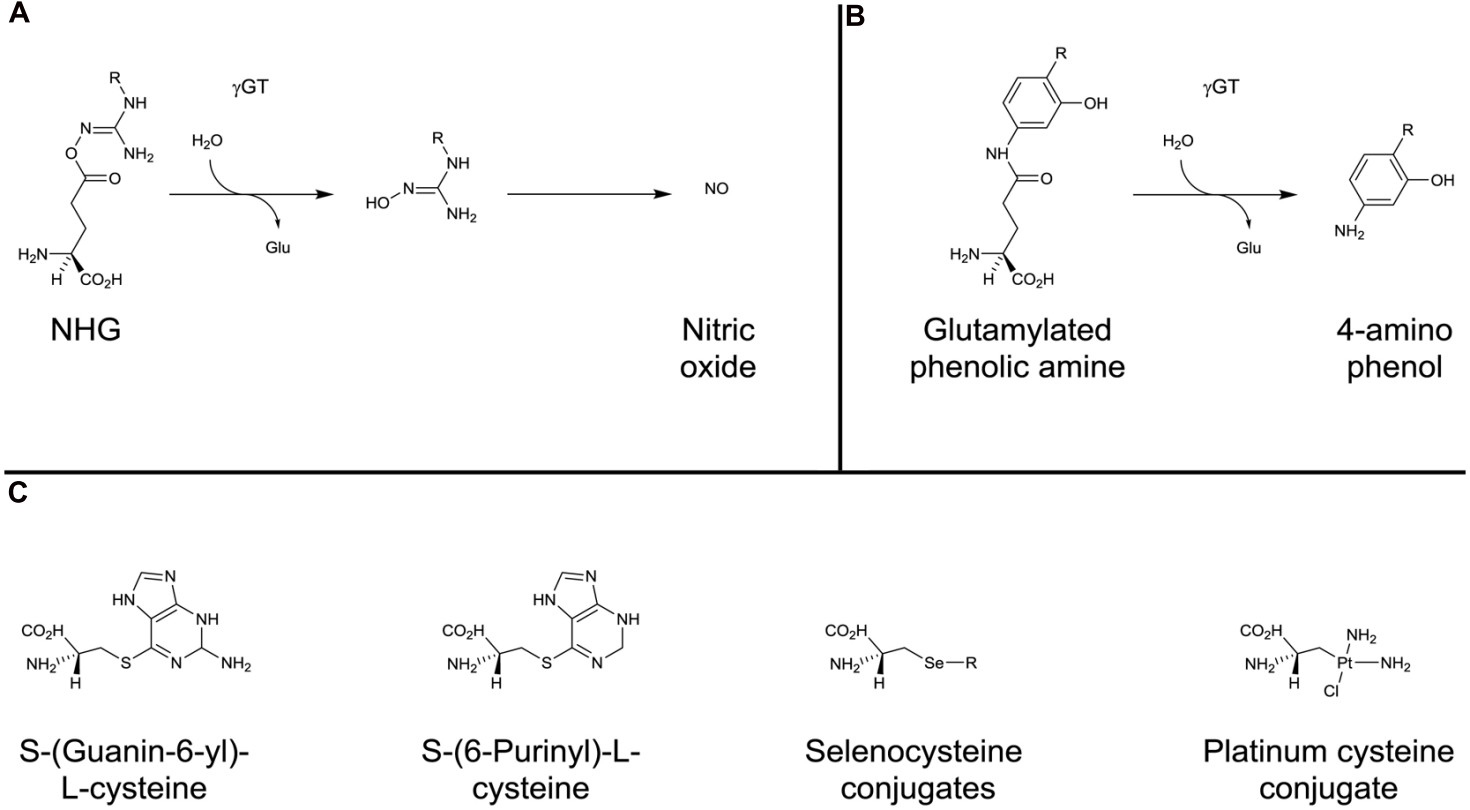
**γ-glutamyl conjugates and β-lyase substrates.** Activation of *N*-hydroxyguanidines **(A)** and glutamylated phenolic amines **(B)** by γGT. **(C)** examples of cysteine- and selenocysteine-conjugates activated by β-elimination catalyzed by β-lyase.

In terms of potential antitumor activity, γ-glutamyl-protected *N*-hydroxyguanidines (NHGs) have been developed to explore the ability to deliver NO to the kidney (**Figure 5A**). The NHGs include *N^w^*-hydroxy-L-arginine, which is an intermediate in the NO synthase synthesis of NO (Zhang et al., 2013b). Whilst promising results were seen, there was a propensity for the potential compounds to cyclize (Zhang et al., 2013a,b) suggesting that NHG conjugation to GSH may be more stable. (Zhang et al., 2013a). As described above for PABA/NO, JS-K, and GSNO, the NHGs, in accordance with γGT expression patterns, contribute to chemotherapy by producing locally high levels of NO which can rapidly react with O_2_^⋅^- to generate the potent oxidant peroxynitrite that, in turn causes extensive cellular damage, including the nitration of protein tyrosine residues (Kirsch et al., 2001).

Glutamylated phenolic amine compounds have been shown to be activated by γGT (**Figure 5B**). γ-L-glutaminyl-4-hydroxybenzene and γ-L-glutaminyl-4-iodobenzene have been demonstrated to be activated by tyrosinase (Prezioso et al., 1993) and γGT (Prezioso et al., 1994a). Both compounds were shown to have greater cytotoxicity in melanoma cell lines when γGT activity was not inhibited (Prezioso et al., 1994a).

#### γGT-activated prodrugs and potential nephrotoxicity

Gamma-glutamyl transferase being the initial and limiting step in γGT-activated prodrugs processing and because γGT is highly expressed on the luminal surface of the proximal tubules in the kidney, potential nephrotoxicity could have been a limitation in the usage of this class of compound when administered systematically. In the case of GSAO, a reversible renal toxicity described in preclinical studies at the maximal tolerated dose was not observed in human during clinical Phase I (Horsley et al., 2013). Similarly, Darinaparsin, when employed in patients (Tsimberidou et al., 2009; Wu et al., 2010; Hosein et al., 2012) didn’t display any toxicity profile related to renal dysfunction at dose-limiting toxicity (DLT). Taken together, these clinical studies indicate that the γGT-activated prodrugs have not necessarily nephrotoxic properties. This type of adverse effects is most likely dependent on the nature of the metabolites produced by the mercapturic acid pathway and particularly if the end products are substrates for cysteine conjugate β-lyases. This enzyme is notably responsible for the nephrotoxicity of cisplatin (see below). However, recent studies demonstrated that the modulation of renal GSH content could prevent such toxicity (Firdous and Kuttan, 2012; Sener et al., 2012; Ghosh et al., 2013).

## β-LYASE AND GENERATION OF CYTOTOXIC PRODUCTS

Cysteine conjugates produced by dipeptidases or amino peptidases following the GST and γGT steps, are transformed by cysteine conjugate β-lyases present in the cytosol and mitochondria. The β-lyases catalyze the β-elimination of various L-cysteine *S*-conjugates to the corresponding thiols (Cooper, 1998). The thiols released can be either toxic or pharmacologically active for the cells responsible for their production (Commandeur et al., 1995). Due to the high expression of cysteine conjugate β-lyase in the proximal tubule of the kidney, administration of cysteine-*S*-conjugates of haloalkenes to rodents results in selective nephrotoxicity (Stevens et al., 1989; Hayden and Stevens, 1990).

The specific localization along with intense expression of these enzymes in renal carcinoma has led to the design of kidney-selective prodrugs that bypass earlier steps of the mercapturic acid pathway (GST, γGT, and peptidase): these compounds include *S*-(guanin-6-yl)-L-cysteine (Elfarra et al., 1995), *S*-(6-Purinyl)-L-cysteine (Hwang and Elfarra, 1991; Lash et al., 1997) and selenocysteine-conjugates (Commandeur et al., 2000; **Figure 5C**). The potential of these types of compounds as kidney-selective antitumor prodrugs has been demonstrated in tissue distribution experiments. However, because of a lower ratio of kidney/liver β-lyase activity in human than rodents, kidney selectivity remains to be confirmed.

Cisplatin is one of the most effective anticancer agents in the treatment of solid tumors, including, breast, testicular, and ovarian cancer. The drug binds DNA, which is toxic to dividing tumor cells. However, therapy with cisplatin is notably limited by a toxicity mechanism in the non-dividing proximal tubule cells of the kidney that is distinct from DNA cross-linking. It was demonstrated that cysteine *S*-conjugate β-lyase highly expressed in the kidney was responsible for the observed nephrotoxicity (Zhang and Hanigan, 2003; Townsend et al., 2003). Even if cysteine *S*-conjugate β-lyase is responsible for the synthesis of the highly reactive and cytotoxic thiol version of cisplatin (**Figure 1**), the activation of cisplatin into a nephrotoxin relies on the three previous steps of the classical mercapturic acid pathway involving GSTs (GSH conjugation), γGT (production of CisPt-cys-glu) and dipeptidase (production of CisPt-cys; **Figure 5C**). Indeed, the inhibition of γGT or cysteine *S*-conjugate β-lyase (both highly expressed in proximal tubule cells of the kidney) blocks the nephrotoxicity of cisplatin in mice (Townsend et al., 2003). In this process, the action of γGT is a key and limiting step as it transformed the essentially membrane impermeable CisPt-GSH into a membrane permeable CisPt-cys-glu that can be taken up by the cells.

## CONCLUSION

The traditional approach to identify potential cancer therapies has been to identify a drug that helps the greatest number of patients. In doing this, the potential of therapies that have a significant impact on a small subset is lost within the larger sample population. The current trend to target specific abnormalities in cancer allows for the development of predictive markers and companion tests to identify which patients have a particular tumor characteristic. From this stems the ability to identify the patient population that will (or will not) respond to the drug. Developing new therapies with the target population in mind will facilitate the development of drugs that will induce a greater tumor response for individual patients. For this to occur, a marker that predicts patient response to the drug is essential.

This review recapitulates the different drug strategies involving GSH-conjugates, and the enzymes of the mercapturic acid pathway, employed over the last twenty years. Amongst the long list of compounds investigated, a limited number of GSH-conjugates have recently progressed towards clinical trials for the treatment of cancer patients. Both GST-activated nitrogen mustard (TLK286) and γGT-activated arsenic-based (GSAO and Darinaparsin) prodrugs have displayed promising activities along with safe profiles.

Given the complexity of mechanisms involved in drug resistance and survival pathways of tumor cells, combination strategies of GSH-conjugates with conventional chemotherapy or targeted drugs should provide very interesting perspectives for the treatment of drug resistant tumors. This approach has already been explored successfully with TLK286 when combined with pegylated liposomal doxorubicin (Kavanagh et al., 2010; Vergote et al., 2010) or with carboplatin plus paclitaxel (Sequist et al., 2009). Moreover, knowing that the combinations of arsenic trioxide with targeted drugs such as mTOR (Guilbert et al., 2013; Iwanami et al., 2013), EGFR (Noh et al., 2010; Kryeziu et al., 2013), or proteasome inhibitors (Jung et al., 2012) demonstrate potent profiles on a variety of malignancies, it would be of great interest to investigate the antitumor properties of such inhibitors in combination with the arsenical-based GSH-conjugates GSAO and Darinaparsin.

Increased GST and/or γGT tumor expression, generally considered as a bad prognosis marker (associated with drug resistance and disease progression), and could potentially be used as a predictive marker, allowing doctors, after biopsy, to identify which patients will respond best to GST or γGT-activated prodrugs. Predictive biomarkers provide a number of benefits, including: reduced unnecessary treatment; reduced quantity of adverse events; reduced drug attrition rates; improved therapeutic benefit; and better control of medical costs (La Thangue and Kerr, 2011). Whilst taking the time to test for GST and/or γGT tumor expression may slightly delay treatment, it will ensure that GSH-conjugates anticancer prodrugs will be administered to the patients that are more likely to respond.

## Conflict of Interest Statement

The authors declare that the research was conducted in the absence of any commercial or financial relationships that could be construed as a potential conflict of interest.

## ABBREVIATIONS

6-MP: 6-mercaptopurine
6-TG: 6-thioguanine (6-mercaptopurine and 6-thioguanine)
CAO: 4-[N-(S-cysteinylacetyl)amino]phenylarsonous acid
cAVTP: cis-6-(2-acetylvinylthio)-purine
CPIC: p-chlorophenyl isocyanate
DMAC: dimethylarsino-cysteine
DMACG: dimethylarsino-cysteinylglycine
EGFR: epidermal growth factor receptor
GSH: glutathione
GCAO: 4-[N-(S-cysteinylglycylacetyl) amino] phenylarsonous acid
γGT: gamma-glutamyl transferase
GSAO: [4-(N-(S-glutathionylacetyl) amino)phenylarsonous acid]
GS-cAVTP: glutathione s-cis-6-(2-acetylvinylthio)-purine
Glu: glutamate
Gly: glycine
GS-tAVTG: glutathione s-trans-6-(2-acetylvinylthio)-guanine
PENAO: 4-(N-(S-penicillaminylacetyl)-amino)phenylarsonous acid
SCPG: S-(N-pchlorophenylcarbamoyl) glutathione
tAVTG: trans-6-(2-acetylvinylthio)-guanine.
